# Comparative Outcomes of the Two Types of Sacral Extradural Spinal Meningeal Cysts Using Different Operation Methods: A Prospective Clinical Study

**DOI:** 10.1371/journal.pone.0083964

**Published:** 2013-12-26

**Authors:** Jian-jun Sun, Zhen-yu Wang, Mario Teo, Zhen-dong Li, Hai-bo Wu, Ru-yu Yen, Mei Zheng, Qing Chang, Isabelle Yisha Liu

**Affiliations:** 1 Department of Neurosurgery, Peking University Third Hospital, Peking University, Beijing, China; 2 Department of Neurosurgery, Institute of Neurological Science, Glasgow, United Kingdom; 3 Department of Neuroradiology, Peking University Third Hospital, Peking University, Beijing, China; 4 Department of General Surgery, Peking University Third Hospital, Peking University, Beijing, China; 5 Department of Neurology, Peking University Third Hospital, Peking University, Beijing, China; 6 Department of Pathology, Peking University Third Hospital, Peking University, Beijing, China; 7 Department of Head and Neck Surgery, UCLA Medical Center, Los Angeles, California, United States of America; University of Toronto, Canada

## Abstract

This prospective study compares different clinical characteristics and outcomes of patients with two types of sacral extradural spinal meningeal cysts (SESMC) undergoing different means of surgical excision. Using the relationship between the cysts and spinal nerve roots fibers (SNRF) as seen under microscope, SESMCs were divided into two types: cysts with SNRF known as Tarlov cysts and cysts without. The surgical methods were tailored to the different types of SESMCs. The improved Japanese Orthopedic Association (IJOA) scoring system was used to evaluate preoperative and postoperative neurological function of the patients. Preoperative IJOA scores were 18.5±1.73, and postoperative IJOA scores were 19.6±0.78. The difference between preoperative and postoperative IJOA scores was statistically significant (t = -4.52, *p* = 0.0001), with a significant improvement in neurological function after surgery. Among the improvements in neurological functions, the most significant was sensation (z=-2.74, *p*=0.006), followed by bowel/bladder function (z=-2.50, *p*=0.01). There was a statistically significant association between the types of SESMC and the number (F=12.57, *p*=0.001) and maximum diameter (F=8.08, *p*=0.006) of the cysts. SESMC with SNRF are often multiple and small, while cysts without SNRF tend to be solitary and large. We advocate early surgical intervention for symptomatic SESMCs in view of significant clinical improvement postoperatively.

## Introduction

Sacral extradural spinal meningeal cysts (SESMCs) are extradural meningeal cysts located in the sacral canal. These cysts are commonly an incidental finding on magnetic resonance imaging (MRI), and are usually asymptomatic. According to the classification of sacral spinal canal cysts by Nabors et al [1], SESMCs are divided into two types: those with spinal nerve roots fibers (SNRFs) and those without. SESMCs with SNRFs, also called Tarlov cysts, are characterized by collections of cerebrospinal fluid (CSF) between the endoneurium and perineurium of the nerve root sheath near the dorsal root ganglion [2]. 

Despite advances in diagnosis techniques, the optimal treatment of symptomatic SESMCs remains unclear. Nonsurgical options include lumbar CSF drainage and computed tomography (CT)-guided percutaneous cyst aspiration with or without infusion of fibrin glue, but these do not prevent recurrence of symptomatic cyst [3]. The goals of surgical intervention of symptomatic SESMCs are to relieve nerve irritation and compression, and to stop bone erosion. Neurosurgical techniques for treating symptomatic SESMCs include lumbar-peritoneal shunt placement, simple decompressive laminectomy, partial cyst removal and neck ligation with or without nerve root resection, cyst wall excision, cyst fenestration and cyst shrinkage using bipolar cautery [4]. Although there is no consensus regarding the definitive treatment of symptomatic SESMCs, several previous studies have reported surgical methods have the best long-term outcomes [1-3]. 

Up to now, most studies focused on Tarlov cysts [2-8], rather than both types of SESMCs. Most previous reports describing the management of SESMCs presented either single cases or a small series of no more than 20 case reports [1-8]. In this study, we aimed to identify and treat a larger cohort of patients with SESMCs, prospectively study their clinical outcomes, and to evaluate the treatment of this rare disorder.

There are several theories about the formation and clinical manifestations of Tarlov cysts (SESMCs with SNRFs) [2,9], but none on SESMCs without SNRFs. There are many options for nonsurgical and surgical interventions on SESMCs with SNRFs, each with advantages and disadvantages [2–9]. Most authors advocate surgical intervention for SESMCs with SNRFs [2,6,9], despite the lack of reports on how to treat SESMCs without SNRFs. Until now, the etiology, clinical manifestations and treatments for SESMCs without SNRFs have not been discussed in the literature. 

Regardless of the nomenclature or classification system, the defining feature of Tarlov cysts (SESMCs with SNRFs) is the presence of SNRFs within the cyst wall or cyst cavity. Although CT myelography and MRI imaging are useful tools, the final diagnosis of a Tarlov cyst (as opposed to SESMCs without SNRFs) can only be made intraoperatively under the surgical microscope[3]. This study seeks to investigate the differences in clinical manifestations, surgical techniques, and outcomes between patients with the two different types of SESMCs. 

## Materials and Methods

This is a prospective cohort study using clinical records, operation notes and patients outcome assessment. Approval for the study was obtained from the Research Ethics Board of Peking University Third Hospital. All subjects were unrelated individuals. The patients or guardians provided written informed consent for surgical operation and medical photography as well as inclusion in the prospective cohort clinical study. 

### 1: Patients

From June 2009 to June 2012, a consecutive series of 55 patients diagnosed with symptomatic SESMCs underwent microsurgical treatment. Basic demographics, clinical and radiological data were evaluated. Preoperative neuroimaging including MRI was performed in all cases. The period of symptoms before presentation was defined as the time from the onset of symptoms to the time of presentation, and was recorded in months. 

Among 55 patients, 17 were male (30.9%) and 38 were female (69.1%). Age ranged from 13 to 70 years old, with an average age of 40.4±14.31 years. Average length of hospital stay was 15.8±5.35 days.

### 2: Inclusion and Exclusion Criteria

Patients were included in the study if they met the following criteria: (1) radiological findings consistent with SESMCs and (2) neurological symptoms attributable to SESMCs. Patients were excluded from the study if their symptoms could not be differentiated from lumbar spinal stenosis or lumbar intervertebral disc herniation. 

The patients with SESMCs were divided into two different groups based on whether their cysts contained SNRFs. The clinical data in the different patient groups were collected prospectively by clinicians blind to the SNRF status of the patients. This data was then analyzed using the appropriate statistical methods. 

### 3: Clinical Presentation

The initial presenting symptoms, location of presenting symptoms, and period of symptoms before initial presentation were recorded. The presenting symptoms were categorized into (1) bowel/bladder and sexual dysfunction, (2) lower extremity weakness, (3) lower extremity numbness, (4) pain, (5) tenesmus (6) more than one symptoms. The location of the presenting symptoms was categorized as (1) sacrococcygeal, (2) perineum and external genitalia, (3) low waist, (4) buttocks, (5) legs, or (6) more than one region. 

Abnormalities on neurological examination were classified as sensory dysfunction, lower extremity weakness, and bowel/bladder dysfunction. The Improved Japanese Orthopaedic Association (IJOA) grading system was used to evaluate pre-operative and post-operative neurological function. The IJOA is based on the Japanese Orthopaedic Association (JOA) grading system, with the addition of a score for bowel function as normal, slight dysfunction, severe dysfunction, or incontinent [10]. 

### 4: Surgical Techniques

When neural irritation symptoms occurred in patients with sacral extradural spinal meningeal cysts (SESMCs), and when bone erosion was found in the neuroimaging, surgical intervention was highly recommended for these patients. When sacral extradural spinal meningeal cyst was discovered incidentally, the patient would be kept under yearly surveillance. Surgical intervention would only be carried out if the cyst progressively enlarged, or patient became symptomatic.

**Figure 1:**
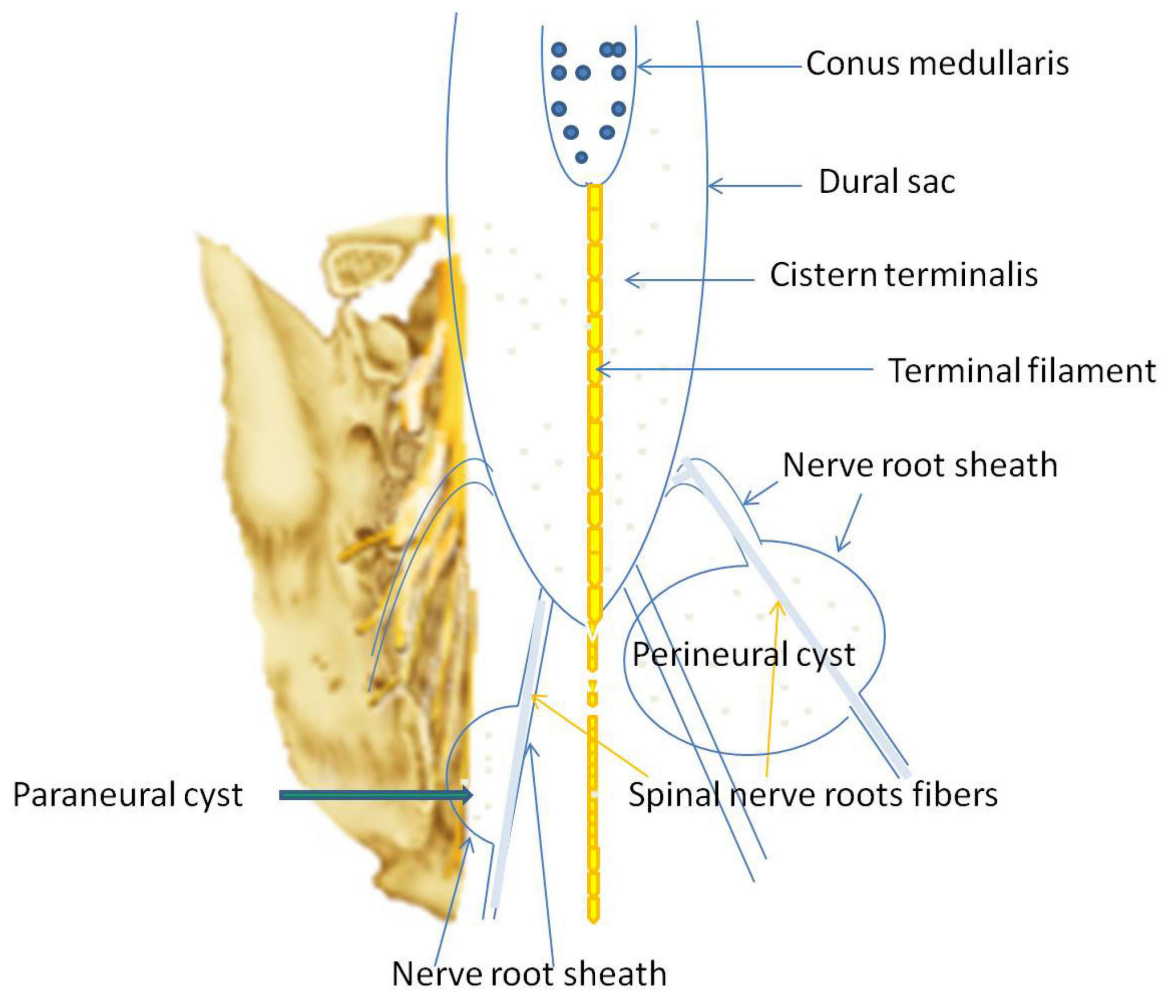
Two types of sacral extradural spinal meningeal cysts with spinal nerve root fibers.

**Figure 2:**
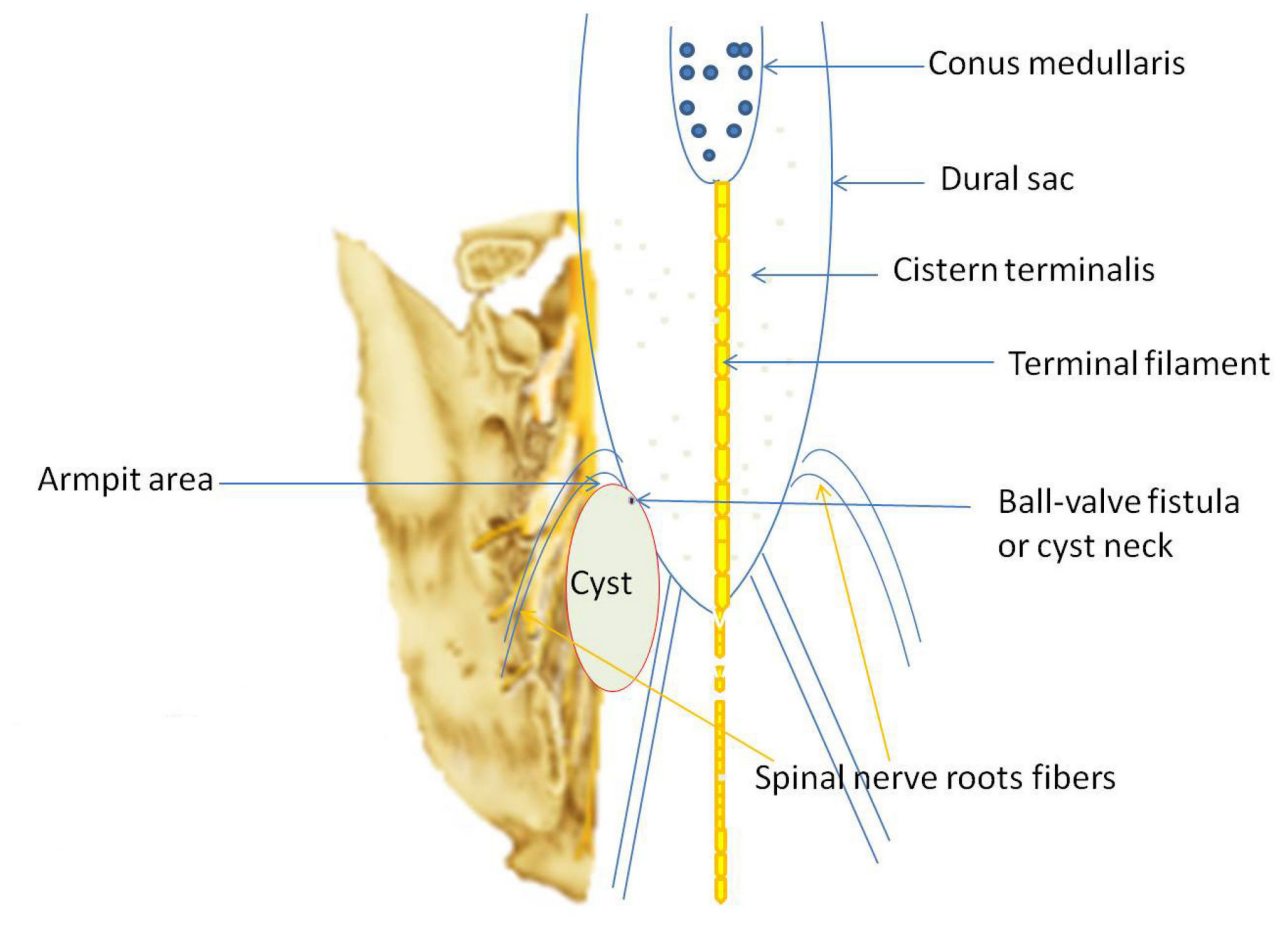
Sacral extradural spinal meningeal cysts origin in the armpit of spinal nerve roots fibers.

**Figure 3:**
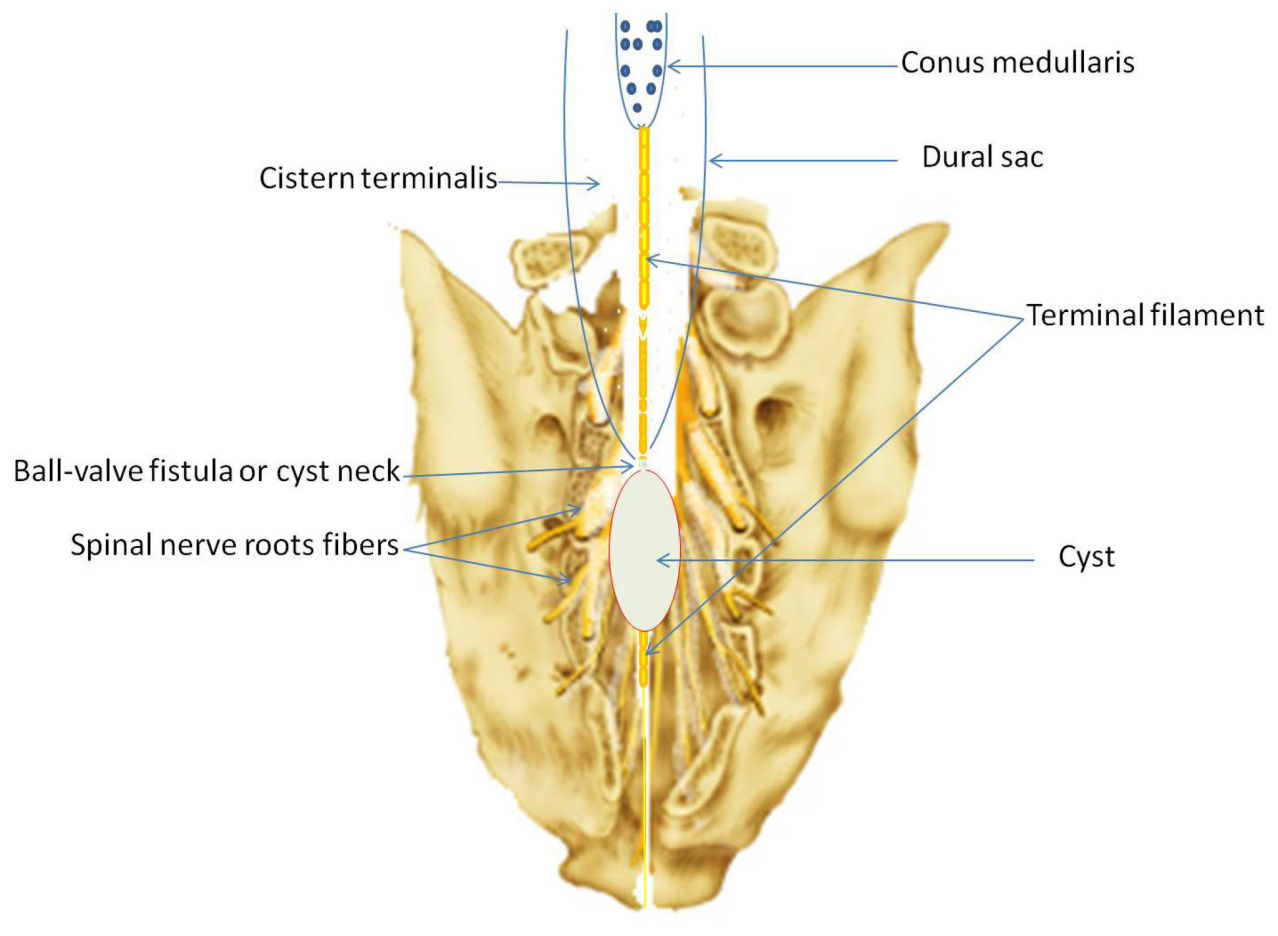
Sacral extradural spinal meningeal cysts origin directly from extremity of terminal pool without spinal nerve roots fibers.

Our operative technique followed the standard procedures for SESMCs surgery. An incision was made from L5 to S3, and the sacral laminae were completely exposed according to the location of SESMCs. Laminectomy was performed with a rongeur, while carefully preserving the integrity of the underlying cyst. The surgical microscope was then brought into the field. The terminal thecal sac was identified and dissected free from the overlying cysts. Each cyst was dissected from surrounding structures to reveal its origin and relationships with SNRFs by the senior authors (ZY Wang, JJ Sun). If the SESMCs were identified as those with SNRFs (Figure 1), the cysts were partially resected and the defect oversewn to prevent CSF leakage from the subarachnoid space and the nerve root sheath reconstructed. Redundant cyst wall was shrunk using bipolar cautery. If the SESMCs were identified as those without SNRFs, which originated in the armpit of SNRFs (Figure 2) or extremity of terminal pool (Figure 3), the neck of cyst was transfixed, ligated and the remaining cyst wall resected distal to the ligation. If the cysts were associated with a tethered cord, then untethering would be performed during the same procedure. Intraoperative neurophysiological monitoring was used to differentiate SNRFs from other tissues. Electrical stimulation was used to verify that no motor nerve fibers were involved. The closure was reinforced with a local muscle flap. 

### 5: Characteristics of SESMCs

Under the surgical microscope, the number, maximum diameter, and types of cyst were identified. If there were multiple cysts in the sacral canal of one patient, the maximum diameter, types of cyst and surgical technique were recorded for the largest one. 

### 6: Postoperative Management and Evaluation

Postoperatively, all patients were kept in the prone position for several days. Wound healing was classified into well-healed, delayed healing, or requiring debridement/suturing. MRI scans were performed two weeks following surgery. Postoperative radiological evaluation of the sacral canal was performed by a neuroradiologist, blinded to the patient’s intraoperative diagnosis, and was classified as complete cyst resolution, residual cyst, or disappearance of cysts but with effusion into the canal cavity. Postoperative IJOA scores were evaluated three months after surgery. 

### 7: Follow-up

All patients were followed up until July 2013 by outpatient clinical visits or by telephone questionnaires. The outcome of each patient was assessed using the IJOA score, and subjective assessment of whether preoperative symptoms remained the same or improved as good outcome, or worsening symptoms as poor outcome.

### 8: Statistical Analysis

Data analysis was performed using SPSS 17.0 (SPSS, Chicago, USA). Data were compared using the two-sample t-test or one way ANOVA test for the parametric data, and the Mann-Whitney or Wilcoxon tests for non-parametric data. Data were expressed as the mean ± standard error. P-values of equal or less than 0.05 were considered statistically significant. 

## Results

### 1: Presentation

The most commonly involved location was multiple regions at initial presentation (40%, 22/55), followed by low waist (20%, 11/55), legs (20%, 11/55), sacrococcygeal (9%, 5/55), buttocks (7%, 4/55) and the perineum and external genitalia (4%, 2/55). The difference in the involved locations for SESMC with or without SNRF at initial presentation was not statistically significant (Z=-1.41, *p*=0.16). The most common presenting symptom was pain (72.8%, 40/55), followed by more than one symptoms (10.9%, 6/55), numbness (9.1%, 5/55), bowel/bladder and sexual dysfunction (3.6%, 2/55), lower extremity weakness (1.8%, 1/55) and tenesmus (1.8%, 1/55). There was a statistically significant difference in the presenting symptoms of the two types of SESMC, with 29/34 (85%) of SESMC with SNRF presenting with pain, compared to 11/21 (52%) of SESMC without SNRF (Z=-1.92, *p*=0.05). The mean interval for initial symptoms to presentations was 39.8±51.55 months. There was a statistically non-significant difference in the duration of symptoms prior to presentation of the two types of SESMC. (Table 1)

**Table 1:** The relation between initiation presenting symptoms and different type of sacral extradural spinal meningeal cysts.

	SESMC with SNRF	SESMC without SNRF	p value
**Number of patients**	34	21	
**Age (median**)	39.9±15.77	41.1±11.89	0.75
**Range**	13~70	14~61	
**Gender (M:F)**	11:23	6:15	0.77
**Duration of symptoms(months)**	36.2±52.78	45.4±50.22	0.53
**Initiation Presenting Symptoms**			0.05
Lower extremity weakness	0	1	
Tenesmus	1	0	
Bowel/bladder and sexual dysfunction	0	2	
Lower extremity numbness	1	4	
More than one symptom	3	3	
Pain	29	11	
**Involved location of initiation symptom**			0.16
Perineum and external genitalia	0	2	
Buttocks	1	3	
Sacrococcygeal region	3	2	
Low waist	8	3	
Legs	5	6	
More than one region	17	5	

### 2: Nerve Functions

The mean preoperative IJOA score was 18.5±1.73, three-month postoperative IJOA score was 19.6±0.78. The differences between preoperative and three-month postoperative IJOA scores was statistically significant (t=-4.52, *p*=0.0001), indicating a significant improvement in neurological function after surgery. The most significant area of improvement in neurological function was sensation (z=-2.74, *p*=0.006), followed by bowel/bladder functions (z=-2.50, *p*=0.01). The changing of lower extremity weakness was not statistically significant (z=-0.91, *p*=0.37). (Table 2)

**Table 2:** The relation between nerve functions and different type of sacral extradural spinal meningeal cysts.

	SESMC with SNRF	SESMC without SNRF	P value
Preoperative sensation dysfunction	14	12	0.25
Preoperative lower extremity weakness	9	9	0.21
Preoperative grading of bowel/bladder			0.14
Normal	27	13	
Slightly dysfunction	4	4	
Severely dysfunction	2	1	
Incontinent	1	3	
Preoperative IJOA scores	18.9±1.22	17.7±2.17	0.02
Postoperative improvement in sensation	28	17	0.82
Postoperative improvement in lower extremity weakness	7	8	0.16
Postoperative grading of bowel/bladder			0.23
Normal	30	16	
Slightly dysfunction	3	3	
Severely dysfunction	1	2	
Incontinent	0	0	
Postoperative IJOA scores	19.6±0.59	19.5±1.03	0.72

### 3: Characteristics of SESMCs and Relative Analysis

**Figure 4:**
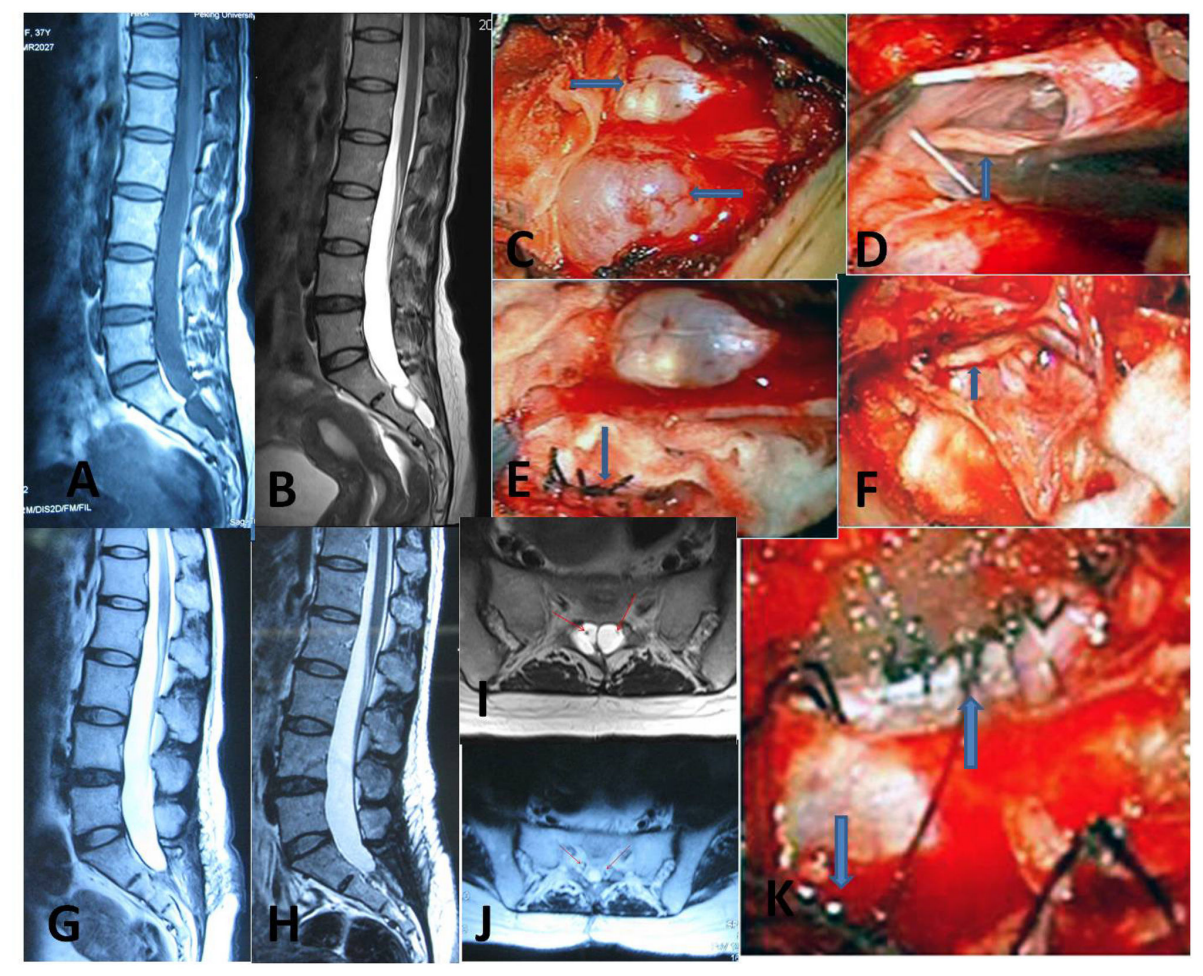
A special case with sacral extradural spinal meningeal cysts with spinal nerve roots fibers. A 38-year-old previously healthy female patient presented with a 40-day history of radiating pain on low waist, buttocks and bilateral legs, which were not relieved by strong analgesia and bed rest. The pain was also exacerbated by posture and exertion. The preoperative IJOA score was 19. A MRI scan showed multiple small cysts in sacral canal. The beadlike cysts showed very low signal intensity on T1-weighted images (**A**), and very high signal intensity on T2-weighted images (**B**). Two adjacent perineural cysts were shown on axial view (**C**, arrow showed the nerve roots). Two cysts were found under microscope intraoperative (**D**, arrow showed the cysts). After opening the cysts, the nerve roots were found inside (E and G, arrow showed the nerve roots). Reconstructed nerve sheaths were performed (F and H, arrow showed the reconstructed nerve sheaths). Two weeks after surgical intervention, no residual cyst was observed in the sacral canal on T2-weighted images (**I**). Two reconstructed nerve sheaths showed on axial view (**J**, arrow showed the nerve root). One year later, no recurrent cyst was found on T2-weighted images (**K**). She also experienced complete resolution of her preoperative radiating pains.

**Figure 5:**
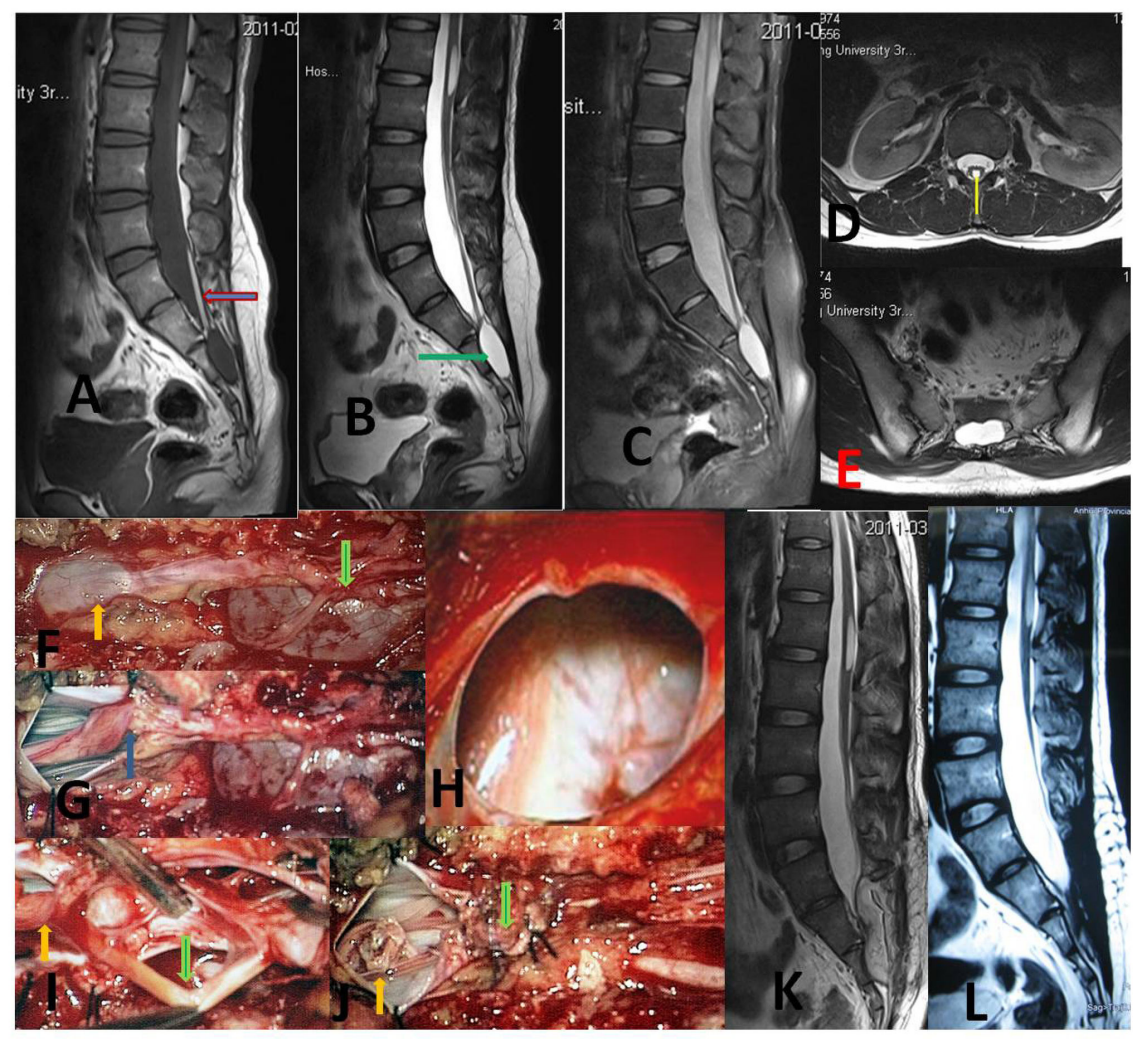
A special case with sacral extradural spinal meningeal cysts without spinal nerve roots fibers. A 30-year-old previously healthy male patient presented with a 1-year history of numbness from left leg to buttock, and sexual dysfunction. The preoperative IJOA score was 18. A MRI scan showed single large cyst in sacral canal. The single cyst combined with tethered cord was shown on T1-weighted images (**A**, an arrow showed thickening inner terminal filament), and T2-weighted images (**B**, an arrow showed the high signal cyst). The inner terminal filament showed slight low signal intensity on T1 -weighted fat-suppression view (**C**). The thickened inner terminal filament showed high signal intensity on T2-weighted axial view (**D**, yellow arrow). SESMC without sacral nerve roots fibers was showed on T2-weighted axial view (**E**). Terminal thecal sac and cyst were shown under microscope intraoperative (**F**, yellow arrow showed terminal thecal sac and green arrow showed cyst). After the dura opening, the thickened inner terminal filament (**G**, arrow) was shown. The cyst was opened to confirm without nerve root inside (**H**). During dissection the distal cyst wall, inner (**I**, yellow arrow) and outer (green arrow) terminal filament were shown together. The neck of cyst was transfixed, ligated (**J**, green arrow) and untethering (yellow arrow) was also performed during the same procedure. No residual cyst was seen in the sacral canal two weeks after surgical intervention, as shown on T2-weighted images (**K**). No cyst recurrence was observed at one year follow up on MRI T2-weighted images (**L**). He also experienced slow improvement of preoperative numbness and sexual dysfunction.

34 patients had SESMC with SNRFs, and 21 patients had. cysts without associated SNRFs. The maximum diameter of every SESMC was larger than 1.5 cm. The mean number of SESMCs was 1.5±0.72. The mean maximum diameter was 3.9±2.17 cm. The average number (F=12.57, *p*=0.001) and maximum diameter (F=8.08, *p*=0.005) of cysts were influenced by the types of SESMCs. Multiple and smaller cysts were relatively more common in the cyst with SNRFs, while single and larger cysts were relatively more common in the cyst without SNRFs. (Table 3, Figure 4-5) 

**Table 3:** Characteristics of sacral extradural spinal meningeal cysts.

	Cysts with nerve roots	Cysts without nerve roots	P value
Maximum diameter	3.3±1.61	4.9±2.60	0.005
Number of cysts			0.001
1	16	19	
2	11	2	
3	7	0	

**Figure 6:**
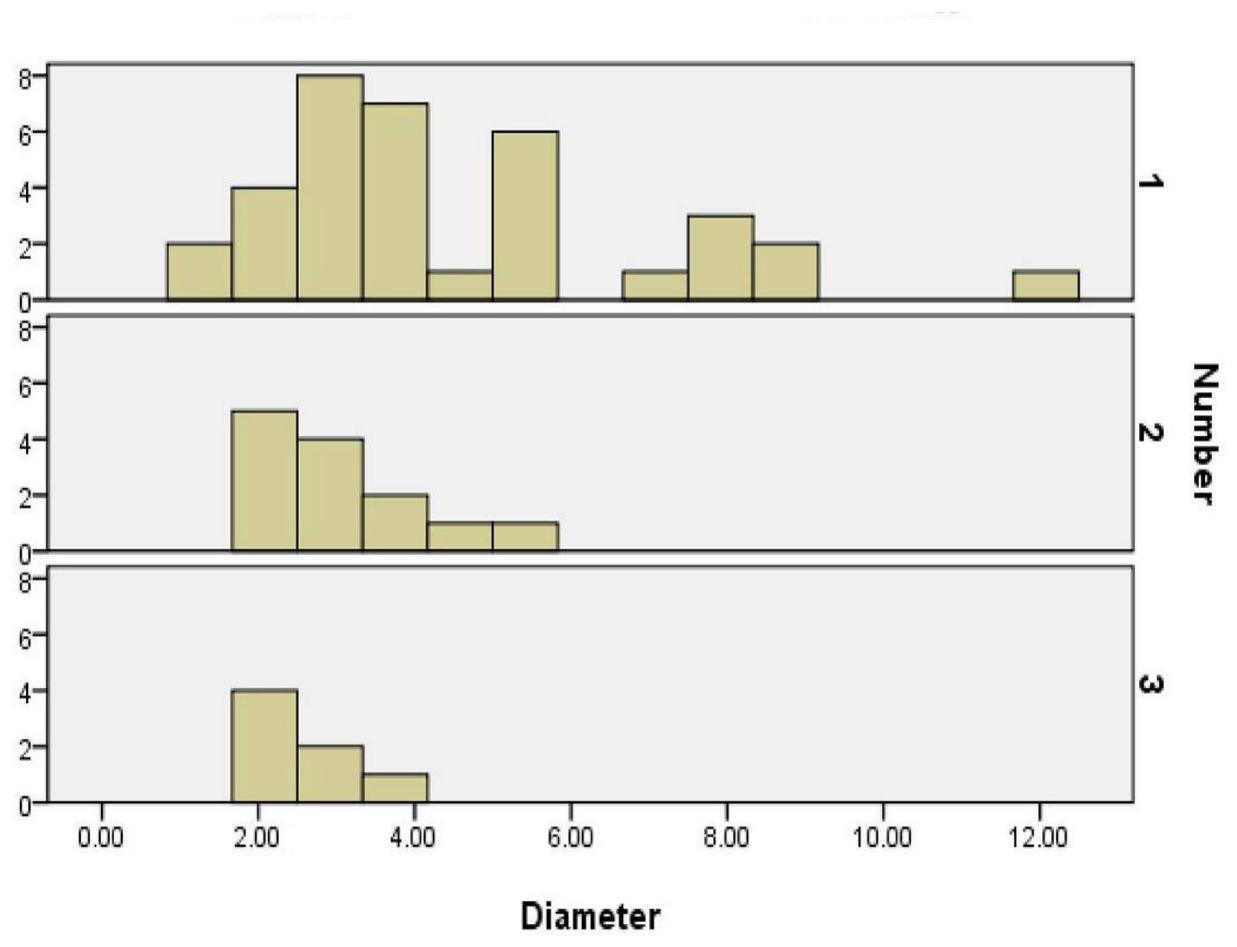
The relation between number and maximum diameter of cysts.

The mean maximum diameter for single sacral cyst was 4.4±2.46 cm. For patients with two sacral cysts, the mean maximum diameter was 3.0±1.05 cm. For patients with three sacral cysts, the mean maximum diameter was 2.6±0.56 cm. The negative correlation between the diameter and the number of cysts was statistically significant (F=3.89, *p*=0.03). (Figure 6)

**Figure 7:**
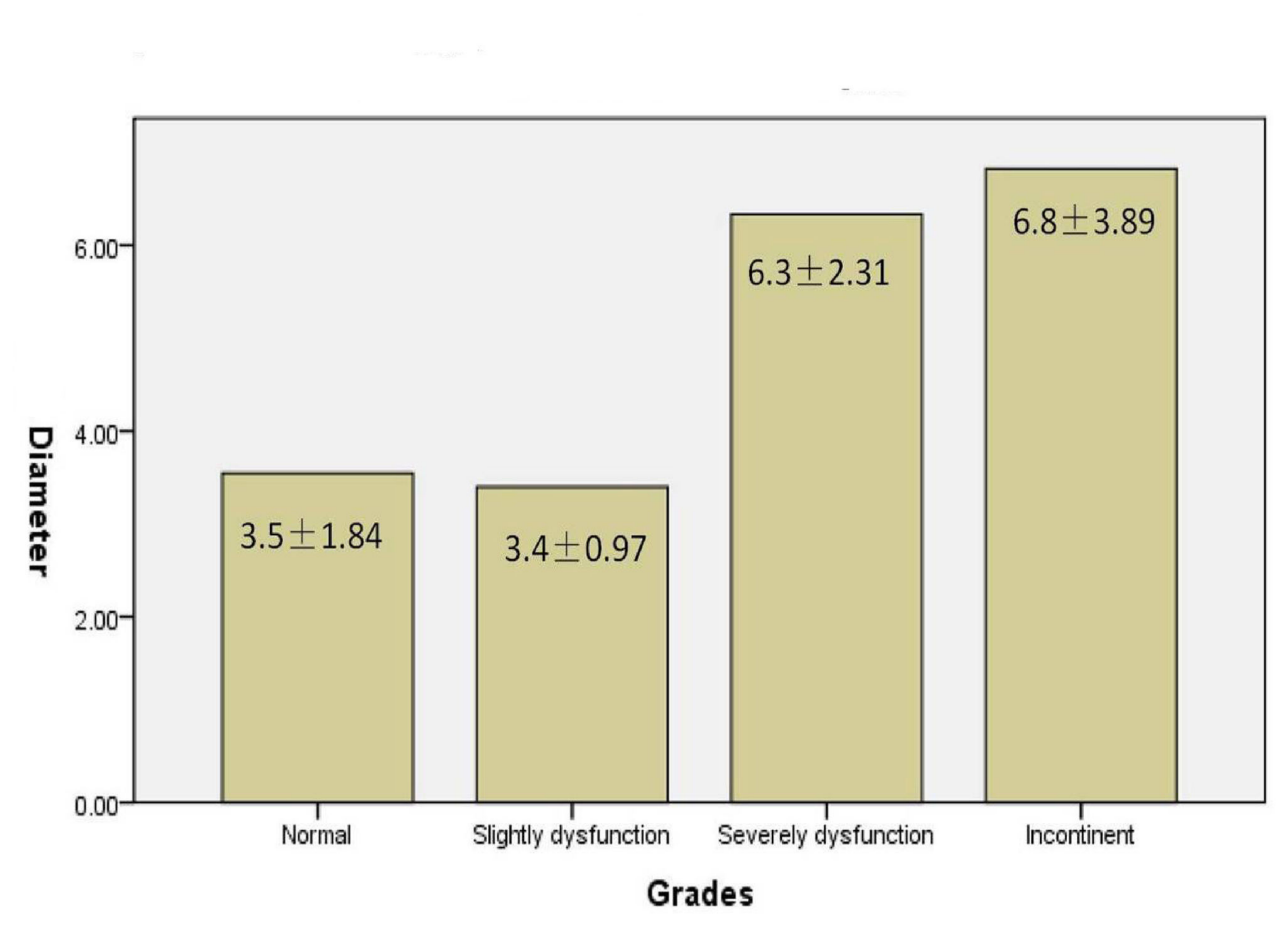
The relation between maximum diameter of cysts and grading of sphincteric function of patients.

The classification of bowel/bladder function was correlative to the maximum diameter of cysts. Patients with either normal or slight bladder/bowel dysfunctions had smaller cysts compared to those who suffered severe sphinteric dysfunction or incontinence (F=5.14, *p*=0.003,). The maximum diameter of cysts of patients graded as either normal or slight dysfunction was mostly less than 6 cm, while the maximum-diameter for cysts of patients graded as either severe dysfunction or incontinence were mostly more than 8 cm. The bigger cyst caused more neurological dysfunction. (Figure 7)

### 4: Postoperative Course

The mean duration of prone position after surgery was 4.9±2.16 days. Wound healing was classified as well healed in 47 cases (86%), delayed healing in 4 cases (7%), and 4 cases (7%) required further debridement and resuturing. Among the four patients requiring debridement/suturing, two (3.6%) patients needed another open operation due to worsening pseudomeningocoele two-month after the first operation. During the second revision operation, potential communicating fistulae were checked, identified and oversewn or transfixed. Prior to final closure, a valsalva maneuver and head tilt were performed to ensure the integrity of the repair, and the wound reinforced with a large muscle flap.

Postoperative radiographic evaluation of the sacral canal was classified as complete cyst resolution in 30 patients (55%), residual small cyst in 9 patients (16%), and disappearance of cyst but with effusion into the canal cavity in 16 patients (29%). The patients with large effusion into the canal cavity and residual small cysts were followed-up closely after the operation. 

There was no difference in the postopoperative outcome and IJOA score for the 2 types of SESMC. Among 15 (27.3%) patients with bowel/bladder dysfunction preoperatively, nine (16.4%) patients did not experience improvement of their sphincteric function, and this was the major factor that cause poor outcome in the longer term follow up (χ^2^=22.68, p=0.0001). Three (5.5%) patients with poor prognosis had severe bowel/bladder dysfunctions. Among them, one (1.8%) patient had severe bowel/bladder dysfunction, aggravation of sensory dysfunction, and leg weakness. Worse wound healing and severe bowel/bladder dysfunction resulted in lower postoperative IJOA scores after three months. 

### 5: Follow-up and Prognosis

The mean follow-up period was 27.5±12.55 months (range 12~48 months). The outcome was classified as good in 52 patients (95%) and poor in 3 cases (5%). Postoperative bowel/bladder functions (normal in 46 cases, abnormal in 9 cases) was the major factor correlating to patient outcome (χ^2^=22.68, *p*=0.0001). The patient with poor prognosis had severe bowel/bladder dysfunctions. 

## Discussion

Two months after being born, as the development of spinal cord and spine progresses, relative ascending of the spinal cord takes place [11]. Conus medullaris ascends to T12~L2 vertebral level. Dural sac terminated in the S2 level. Cistern terminalis accumulated with CSF in subdural space subjacent conus medullaris. The whole structure including cistern terminalis and terminal thecal sac is like a “funnel”. Cistern terminalis is like “funnel’s body”, and terminal thecal sac is like “funnel’s neck”. Inner terminal filament ended in S2 level. Outer terminal filament and cauda equina nerve roots cross thecal sac and adhere to coccygeal vertebral. In summary, capacious space of sacral canal is the anatomical foundation for SESMCs. 

Upright walking is a uniquely human behavior, and is the foundation of human civilization. The hydrostatic pressure of cistern terminalis, the terminal thecal sac, is higher when standing than in other positions. Some individuals may have congenital weakness of the dural diverticulum or nerve sheath. Similar to hemodynamic mechanism of aneurysms formation, years of hydrostatic pressure may cause the weak wall to gradually expand outwards into the sacral space. Upright walking therefore underlies the formation of SESMCs, as advocated by other authors [1-11]. 

Enlargement of SESMCs may be caused by a ball-valve effect, congenital abnormalities, connective tissue disorders, and nerve root sheath duplications. The CSF enters cysts with systolic pulsation or with standing and walking, but unable to exit through the same portal during diastole or motionless activity [12]. A ball-valve effect of the cyst neck results in a gradual increase in the size of the cyst [13]. This ball-valve effect is the usual mechanism for initiation and development of SESMCs. In addition, similar to extradural spinal arachnoid cysts [14], the formation of SESMC may be due to CSF secretion too. It was found that some cysts were covered by arachnoid linings intraoperative, however, more work needs to be done in this field to establish the actual pathogenesis of SESMCs.

During development and enlargement of SESMCs, the adjacent nerve roots fibers are irritated and displaced, causing a variety of symptoms. The most common initial symptom is pain, and multiple anatomical regions are most commonly involved at the time of initial presentation. Because of the relatively weak dorsal and lateral walls of the sacral canal, the enlarging cysts extend in the dorsal, lateral and downward directions. As the mass enlarges, sensory nerve root filaments are stretched over the periphery of the lesion or are compressed against other adjacent nerve roots, causing pain or other sensory disturbances at multiple levels [2,15]. 

It has been found that certain types of SESMCs significantly influenced the number and the maximum diameter of cysts. SESMCs with SNRFs often present with multiple smaller cysts. However, SESMCs without SNRFs often present with single larger cysts. SESMCs with SNRFs (including paraneurium and perineurium) extending around the circumference of the nerve may enlarge to compress the neighboring nerve roots. Patients may begin to feel pain or paresthesiae early, and even very small cysts with SNRFs can therefore be diagnosed early (a case shown in Figure 4). 

SESMCs without SNRFs often locate at the armpit of SNRFs or the extremity of the terminal pool. These patients experienced relative long-term asymptomatic interval (a case shown in Figure 5). However, the larger cyst creates worse nerve dysfunction. Preoperative IJOA scores of patients with cysts without SNRFs are lower than patients with cysts with SNRFs. Although compression of involved nerve roots is released by surgical intervention, nerve function could not recover quickly. So the patients with cysts without SNRFs need a longer time or more hospitalized days for recovery, than patients with cysts with SNRFs. 

Patients with SESMCs with SNRFs might experience various types of pain, and these are varied and related to their sizes and locations. Patients may complain about low back pain, leg pain and sensory disturbances, such as paresthesia or hypoesthesia. Specific radicular pain may result from distortion or compression of the nerve root [2,13]. Pain is usually intermittent and is most frequently exacerbated by standing, walking and coughing [16]. When SESMCs without SNRFs enlarged enough, pressure on adjacent sensitive structures, such as the periosteum and the joint capsule, may cause local or referred pain. Slipman CW et al [17] described a patient suffering from abdominal pain, because the cysts eroded through her sacrum and, by sheer force of their large size, stimulated the retroperitoneal structures. Bowel and bladder dysfunction may be secondary to the disturbance of the sacral parasympathetic flow to the gastrointestinal tract [3]. 

It is generally accepted that asymptomatic SESMCs do not require surgical intervention [1-18]. When these lesions are symptomatic, the goals of surgical intervention are to relieve nerve irritation and compression, and to stop bone erosion. Even in small SESMCs with SNRFs, nerve root irritation can cause severe pain that cannot be relieved by bed rest or strong analgesic and may require urgent surgical intervention. Such symptoms of continuous nerve root irritation may result from a large increase in the volume of CSF in the cyst over a short period of time, which sharply increase the hydrostatic pressure in the cyst resulting in continuous irritation of the surrounding nerve root fibers. Opening and partial resection of the cyst relieves compression of nerve roots and irritation by hydrostatic pressure. Sensory and bowel/bladder dysfunctions are also relieved.

Nevertheless, when the symptom occurred, SESMCs without SNRFs may enlarge enough to fill up the whole sacral space or even to erode bone. The symptoms which are caused by this kind of compression, include dull aches, axial pain or in the longer term. bowel/bladder dysfunctions. Due to the very slow nature of disease progression, most patients understandably were not keen on surgical intervention early on. However, lifelong nerve dysfunction may happen in those with long-term nerve roots compression, which has been demonstrated in our study. Three patients who suffered from cyst without SNRFs complained about abnormal postoperative habits of bowel/bladder and bad prognosis in long-term follow-up. It is therefore emphasized that early surgical intervention should be performed for symptomatic SESMCs. In our study, most patients experienced significant improvement in their neurological function after surgery. The most significant area of improvement in neurological function was sensation, followed by sphincteric function. 

There is still no consensus regarding the appropriate surgical techniques for treating different types of SESMCs. Jung KT [18] et al suggested that conservative treatments such as epidural block would be useful for patients with perineural cysts. Patel MR [19] et al proposed percutaneous drainage and fibrin glue infusion for the treatment of meningeal cysts of sacral spine. However, Voyadzis [9] et al did not recommend percutaneous drainage because of the potential for unexpected nerve root injury. In our experience, fibrin glue infusion may result in pulling of the involved nerve root fibers by the hardened glue. The symptoms of nerve irritation are not relieved, and may even be aggravated by the fibrin glue. When cysts without involved nerve roots fibers are identified under the microscope, use of fibrin glue to seal the fistula may be considered. Smith ZA [20] et al recommended a surgical clip to close the connection between the thecal sac and the cyst as a unique method for preventing recurrence. Tanaka M [13] et al introduced microsurgical treatment consisting of cyst excision combined with direct closure of the dura and plication of the cyst wall. He considered that simple resection or clipping was a hazard to the sacral nerve in cyst wall. Xu IQ [21] et al reported favorable results after microsurgical cyst fenestration and imbrication. 

In our opinions, in order to prevent patients from lifelong nerve dysfunction, early surgical intervention should be performed for symptomatic SESMCs. It is advocated that redundant cyst walls should be resected, communicating fistulae overlap sewn to prevent cysts recurrence, and sacrificing the entire nerve root should be avoided. Based on this principle, if the SESMCs are identified with SNRFs, the redundant cyst wall should be removed, and the communicating fistulae should be oversewn to prevent leakage of CSF from the subarachnoid space and to reconstruct the nerve root sheath. If the SESMCs are identified as those without SNRFs, which originate in the armpit of sacral nerve root fibers or the extremity of terminal pool, the fistulae neck of cyst should be transfixed, ligated and the remaining cyst wall should be excised distal to the ligation. 

It is emphasized that postoperative CSF leakage and cyst recurrence should be avoided by careful microscopic surgical techniques. All communicating fistulas should be identified and transfixed by ligation or oversewn, and a Valsalva maneuver should be performed to ensure that there is no residual leakage. Even with appropriate operative techniques, residual small cysts cannot be completely avoided. Further research is needed to develop surgical techniques and improve treatment. Resection of giant cysts that have developed over a long period of time results in a giant cavity in the sacral canal. As the relatively weak sacrococcygeal muscles and soft tissues cannot effectively fill this cavity, pseudomeningocoele in the canal cavity after surgical intervention is unavoidable. To reduce this effusion in the canal cavity, it is necessary to stay prone in the bed for several days. There is less fluid exudation if the hydrostatic pressure in the cisterna terminalis is kept stable with this positioning. We also used moderate sacral wound compression with a 1 kg clean sandbag, and this resulted in less pseudomeningocoele.

## Conclusion

Patients with SESMCs with SNRF tend to present with pain, while those with SESMC without SNRF tend to present with neurological deficit. Radiologically, SESMCs with SNRFs often have multiple smaller cysts, whereas SESMCs without SNRFs often present with single larger cysts. Early surgical intervention should be performed for symptomatic SESMCs. Most patients experienced significant improvement in their neurological function after surgery. The most significant area of improvement in neurological function was sensation, followed by bowel/bladder function.
